# A Text-book of Practical Medicine

**Published:** 1885-01

**Authors:** 


### Book Reviews.

A Text-book of Practical Medicine—Designed for the use of students and practitioners of medicine, by Alfred L. Loomis, M. D., LL.D., Professor of Practical Medicine in the Medical Department of the University of the city of New York, Visiting physician to Bellevue Hospital, etc, with two hundred and eleven illustrations. William Wood & Co., New York: 1884.

In the December number of our Journal we acknowledged the receipt from the publishers of a copy of the above named book. It is an attempt to comprise, within reasonable limits, a single volume of 1,000 pages, a clear account of the present status of practical medicine in America.

It was a book much needed, and a careful examination of it satisfies us that the author has been eminently successful in its preparation. Covering so vast a field, condensation was a necessity, and being conceded facts, old or new, are stated with clearness and perspicuity, and tedious discussions of unsettled questions or conflicting opinions occupy but little space.

No classification of the subject embraced in this work has ever been proposed which met with universal approval. The one adopted is that which it has been the author's custom to follow in teaching, and claims to be based on our present knowledge of the etiology of disease.

In the compilation of a text-book originality is not to be expected. Truth is to be preferred to novelty; the greatest part of it must consist of old and familiar aphorisms. But the diligent study of morbid phenomena and the impartial analysis of therapeutic effects, everywhere intelligently conducted, are evolving new facts and leading to better methods of cure or treatment. The great labor of the compiler is to scrutinize, classify and arrange the new and the old, and present them in such manner that his completed work shall show the progress and actual position of the science at the date of its publication.

The author is to be congratulated upon his success. As a text-book for students it has no superior; to the busy practitioner, in whatever rank of the profession, it is of infinite value. Every chapter of it is replete with interest and instruction, and gives evidence of great care in the unprejudiced presentation of facts or opinions.

Where the entire work merits such high commendation, it is difficult to cite particularly instances of special excellence, or in the space at our command, to quote passages of it which will enable our readers to form a just estimate of its value. The section upon Diseases of the Respiratory Organs deserves profound study; every page of it teems with valuable information, conveyed in clear language and captivating style.

Our readers will, we doubt not, peruse with peculiar pleasure the important observations on—

"*The Climatic Treatment of Phthisis* a subject which has recently received much attention, but it is to be remembered that its usefulness is confined almost exclusively to the first stage of the disease, and that no absolute rules can be laid down in regard to it. It is well known that some consumptives thrive best in a warm, moist air, others in a cool, dry atmosphere; some are most vigorous in winter, others in midsummer. Each year's experience impresses on me the conviction that while climate, more than any other agent, has a controlling influence over phthisical developments,* each case must be carefully analyzed before any definite directions can be given as to the climate best suited to it. Although we know of no climatic conditions which render phthisis a necessity or an impossibility, still there are conditions which are known to be antagonistic to its development as well as those which favor its development. Scarcely twenty years ago the great desideratum was

*Laennec long ago wrote: "Of all the means hitherto recommended for the cure of phthisis, none have been followed more frequently by complete cessation of the disease than change of climate."

thought to be a warm, dry atmosphere, but we now know that a cold climate not only does not hasten, but often arrests phthisical processes. The statement has been made that "the higher the altitude the less prevalent is phthisis," but the altitude at which such immunity exists varies with the latitude and with the idiosyncrasy of the individual.

*Mountains* and *elevated districts* were thought to be beneficial on account of their elevation alone. But recent investigations show that the absence of atmospheric impurities is the chief element, and that the purity of the air is the chief reason that elevated regions are so beneficial in phthisis. Prof. Tyndall's experiments are of special interest in this connection.* Organic germs are more abundant in the air in the city than in the country. Rain and ozone free the air from them, the latter by oxidation. *Rain* cleanses the atmosphere of solid particles and purifies it by washing down ammonia and carbonic acid. The presence of ozone in the air is presumptive evidence of its purity. The air of high mountains and plateaux and along the shore cf the ocean is richer in ozone than that of the plains. Prof. Tyndall's experiments show that in early summer, the mountains, and in late summer and fall, the sea-shore have their purest air. The benefit which phthisical patients derive from living near pine-forests has long been known. Turpentine exhaled from pine or hemlock forests converts oxygen into ozone, and thus the air of pine-forests becomes pure. Direct inhalation of ozone has little power over phthisis; hence it is not the ozone but the purity of air it induces that renders the air of certain localities so salubrious. It was formerly thought that resorts where no rain fell for weeks and months were the best suited to phthisical subjects, but experience has taught the reverse. Long-continued rains are certainly unfavorable, but cleansing showers act beneficially. The amount of rainfall is not a sure indication of the amount of moisture

*After boiling, filtering and evaporating a vegetable infusion he hermetically sealed it in flasks, which he transported to the Alps 7,000 feet above sea level. Some of the flasks were opened during transportation, and in these millions of organisms developed in the fluid, while the fluid in the flasks that were opened on the mountain remained free from such organisms. By further experiments he showed that dust-laden air was necessary to the procreation of these organisms, and that they are diffused through the atmosphere, although the air in different localities may be infected in different degrees.

in the air of any region, the latter depending more upon the dampness of the soil. The atmosphere of a region with a loose, porous, sandy soil, through which the water filters, and whose surface dries quickly, is never damp; but hard, compact, rocky or clayey regions that drain but slowly and imperfectly, hold the moisture and cause a dampness which is a strong predisposing cause of phthisis.*

"*Atmospheric temperature* is an important element in the climatic treatment of phthisis. Some patients thrive best in a warm sedative climate, others in a cool, stimulating climate. Extended clinical observation leads one to believe that it is neither the heat nor cold of a certain locality, but the *absence of sudden and frequent changes*, which makes it so beneficial to phthisical invalids.

"*Altitude* is regarded by many at the present time as of more importance than any other natural element. As a rule, the atmosphere at elevations of 1,500 or 1,800 feet is purer than on the plains; yet all high altitudes are not thus pure; experiment has shown the atmosphere of some elevated regions to be impure, and that consumptives on such elevations do badly. Something more than altitude is needed to make a given locality suitable to phthisical subjects. Recent investigations show that the similarity in the composition of sea and mountain air, at certain times of the year, is far greater than was at one time supposed. Mountain air is less dense, less humid and lower in temperature than sea air, but in both we find excess of ozone and freedom from organic impurities. Both sea and mountain air are cooler and less subject to frequent variations in temperature than the air of the plains. A slight diminution in atmospheric pressure produces no palpable changes. But a great diminution (say one-quarter) produces serious disturbances of nutrition, developing a condition which favors rather than retards phthisical developments. The effects of diminished atmospheric pressure vary so greatly in different individuals that no practical deductions can be made.

*Laennec states that the dampness arising from such a condition of soil is one of the most certain developing causes of phthisis, and he makes mention of a locality having such a soil, in which the dampness was so constant and of such a character that two-thirds of the resident population died of phthisis.

"The question arises: will this patient be benefited by sea or by mountain air? Beneke's experiments show that tissue changes take place more rapidly on or by the sea than in the mountains. Hence those in whom the process of tissue-change needs no hastening, and those with exhausted nervous systems, with an overtaxed brain from excessive mental labor or an all-absorbing business, and who still retain considerable muscular power—those should go to the *mountains*. While those past middle life, who have developed phthisis late, who are incapable of much muscular activity, and who therefore require stimulation in order to the production of tissue change—such patients do best in *sea air*. Sea air is better suited than mountain air to those who cannot bear sudden changes of temperature, while the susceptibility to such changes is greatly lessened by mountain air.

"On our own continent is found every variety of climate. Permanent improvement only occurs after a prolonged residence in the place which experience proves best suited to each case. A change of climate should not be made every year. The limited space which can be devoted to the consideration of the localities best suited to the phthisical patients in this and other countries will only allow of mention of the most important ones. Every stage of fibroid phthisis, no matter how far advanced or *where* the fibroid developments began, is benefited in the high altitudes found in Colorado and about the Rocky Mountains. But there is one grave objection to Colorado as a winter refuge: the enormous monthly, and also the diurnal, range of temperature must severely try any invalid. During March, 1880, the thermal range at Denver was eighty-three degrees, and in December, 1876, it amounted to ninety-three degrees—a change in a single month greater than occurs at London in a whole year, and greater than occurs at New York in a whole winter.

"In my experience, *catarrhal phthisis* is not benefited in regions of very high altitudes. It is during, or before, the stage of consolidation that persons with this variety of phthisis are to be benefited by climatic influences, and a careful analysis of each case is important before directions can be given as to the region most likely to suit the special requirements of each case. The patient must not wander around till he hits upon the place which suits him; much valuable time is thus lost. Except in those who are convalescing from some acute lung disease, a sojourn in a southern climate during the winter seems after a time to hasten the degenerative processes. My favorite resorts in the winter, for those recovering from acute pulmonary diseases, are Aiken, S. C., Palatka, Enterprise and Gainesville, Fla., Thomasville, Ga., and Nassau. These localities are also favorable for those in whom there are evident phthisical tendencies, but in whom as yet, no physical evidences of pulmonary consolidation exist. My best results, when the evidences of consolidation were present, have been obtained in those who have stayed from one to three years in mountain regions 1,500 to 2,000 feet above the sea. My most decidedly beneficial and permanent results have been obtained in Asheville, N. C., in New Mexico, and in the Adirondack region of New York State. The temperature, rainfall and surroundings of the latter region are all at variance with preconceived notions of a proper "resort for consumptives," but results are strong in its favor. A camp or tent life in the open air is best for those who can enjoy such life. Excursions and cheerful social intercourse in the open air should always be an object. A dreary spot, even with plenty of ozone and elevation, is not of great material benefit.

"I would advocate sanitariums for the phthisical. Not overcrowded hospitals, but cottages and pavilions in sheltered spots, in appropriate climates, and at a given elevation, where privacy and quiet are possible, and where all shall be supervised by a capable and intelligent physician. Minnesota has a dry, cool, exhilarating climate. Southern California, Georgia and South Carolina have a dry, warm atmosphere. The Bermudas, Bahamas, Florida, Turk's Island, Santa Cruz and St Thomas have a warm, moist and usually healthy climate. The extraordinarily dry belt of country which runs northward from San Antonio, Texas, has begun to endanger the supremacy of Florida as a winter health resort for the consumptive. That this belt offers some climatic advantages for weak lungs over the mild but rather humid air of Florida, cannot be doubted. Nassau, the capital of the Bahamas, is a noted resort, and one that suits most phthisical subjects past middle life ; Matanzas, Cuba, has a dry, warm climate, suitable for a winter home for the enfeebled, but not for those who have developed phthisis. It may be that, for various reasons, a phthisical patient prefers a residence abroad. Dry climates near the sea are Malaga, Riviera, and Algiers. Egypt and South Africa are highly recommended by the English physicians for phthisis. Sea voyages to Australia and New Zealand are recommended in cases of "*hemorrhagic phthisis.*" J. Hughes Bennett finds the lakes of Scotland the best resorts for consumptives in the summer. The Engadine has been strongly advocated by many."

Of great value to Southern physicians, and of especial local interest, is the article on "Continued Malarial Fever," or what is more commonly known as Typho-Malarial Fever. Referring to the book for fuller information, we copy what is said of the etiology and treatment of this disease:

"*Etiology.*—It is difficult to determine the true etiology of this fever. That malarial poison is necessary for its development there can be no question. It is equally certain that some other poison besides malaria is in operation whenever it prevails. This poison is not the specific poison of typhoid fever ; nor are its development and spread in any way connected with the excrements of one suffering from the fever. There are a few facts connected with its development which are now well established:

"*First.*—It is met with only in malarial districts.

"*Second.*—In the majority of instances, when this fever has prevailed, its development has been preceded or attended by marked and easily recognized anti-hygienic conditions, such as overcrowding, bad sewerage, and other conditions favorable to the development of septic poison.

"*Third.*—That it is a *non-contagious disease*, and is never propagated from the affected to the healthy, either directly by personal contagion, or indirectly by morbid excretions.

"*Fourth.*—In its morbid anatomy andsymptomatology it is a combination of malarial and septic fever. The special symptoms and lesions of one or the other of these fevers stamp its character. In large cities in which malarial diseases are prevalent, *sewer gases seem to furnish the septic element which is so essential for its development.* The history of disease in New York City during the past few years furnishes striking examples of the combination of these two poisons in developing a type of fever which must be classed under the head of non specific continued fever, attended by typhoid symptoms and intestinal ulceration.*

"*Treatment.*—The treatment varies with its type. No plan can be presented which will be applicable to all cases.

"The first question which meets us is: cannot the development of this fever be prevented? It has been stated that its development was principally due to three causes—namely, malarial poison, overcrowding and improper diet. In a large proportion of instances it is possible to do away with the last two causes. The overcrowding and the faulty diet may be prevented, and thus the septic poison which gives to this fever its "typhoid" type may be destroyed or its development prevented. The strict observance of hygienic laws in the localities where this fever prevails has in some instances entirely changed the type of the disease. Even after the fever symptoms have been well developed, the removal of patients from anti hygienic surroundings has frequently been attended by the most satisfactory results. When isolated cases of this fever are met with in localities apparently free from such sources of infection, a

*When Dr. Drake (Dis. of Inter. Valley of N. A.) called this disease remitto-typhus, he came nearer than any other observer in giving it a name corresponding to its etiology. That it partakes of remittent characteristics, no one can deny; that it has a septic, a "crowd-poison," a "sewer-gas" a "typhus" character, seems to me to be incontestible, hence "remitto-typhus" would be unobjectionable, were not the word typhus now restricted to the specific, contagious fever of that name, whose poison can only cause one disease, and cannot mingle with, or be modified by any other.

careful search should be instituted in order to find the source of the infection. Defective sewerage and faulty drainage have been found to be fruitful sources of infection.

"The therapeutic measures which may be employed in its treatment vary with its type and the peculiarities of each individual case. There are no specifics. In those cases in which the malarial element predominates, the administration of quinine will in many instances arrest its progress or shorten its duration ; but in those cases in which the septic element predominates, while quinine may act as an antipyretic, it has little power to arrest its progress or to shorten its duration, but it will, in many instances, render the course of the fever milder. In those cases in which the malarial element predominates, which are ushered in by distinct chills, followed by one or two distinct remissions and exacerbations, during the first remission twenty or thirty grains of quinine should be administered, in hourly doses of ten grains each. If it is promptly and freely administered, it seldom fails to produce a beneficial effect ; the febrile exacerbations will not return, or if they do they are less severe, and in a few days entirely disappear. In those cases which begin more insidiously and are developed more gradually, if there is a distinct periodicity to the febrile phenomena, without distinct remission, the administration of quinine may cause the fever to run a milder course. If the first full doses of quinine fail to produce any effect in this class of cases, its administration in moderate doses, perhaps ten grains twice a day, must be continued for several days before it will markedly modify the severity of the fever. In no type of the fever does the quinine exert any specific influence except over the malarial element; the enteric phenomena are either not at all, or only indirectly, modified by the antipyretic power of the drug. Hence, it is apparent that in those cases in which the malarial element is slight, and in which the septic element is prominent, while quinine fails to exercise any controlling influence over the progress of the fever, it will mitigate its severity, and act more powerfully as an antipyretic than it will in any other form of continued fever. Warburg's tincture* in many cases will have a controlling power over the fever when quinine fails.

"It has been claimed by some that arsenic has a specific influence over the fever, and that it exercises a peculiar and most beneficial effect upon the intestinal lesions. There is little doubt but that arsenic, like quinine, acts beneficially in many cases of the malarial type of this fever; but unquestionably this beneficial effect is due to its acknowledged power over malarial affections, and not to any specific influence which it has over the fever. As an antiperiodic it is inferior to quinine. Eucalyptus does not act as beneficially in continued malarial fever as in the simpler forms of malarial fever.

---

"*Formula, *Warburg's Tincture:*

Rad. Rhei

P. Aloe Soc.

Rad. Angelica Officinalis, *aa* .......... ℥ iv.

Rad. Helenii

Crocus Hispan.

Sem. Fœniculi

Creta Preparat., *aa* .......... ℥ ij.

Rad Gentian

Rad. Zedoar

P. Cubeb

G. Myrrhæ

G. Camphor

Boletus Laricis, *aa* .......... ℥ i.

Confect, Damocratis* .......... ℥ iv.

Quiniæ Sulph .......... ʒ lxxxij.

Sp. Vini. Rect .......... O xx.

Aquæ Puræ .......... O xij.

Macerate, in a water bath, twelve hours, express and filter.

*Confectio Damocratis:

Cinnamon .......... *fourteen grams*
Myrrh .......... *eleven* "
White Agaric, Spikenard, Ginger, Spanish Saffron, Treacle, Mustard Seed, Frankincense, and Chian Turpentine, each .......... *ten* "
Camel's Hay, Costus Arabacus, Zeodary, Indian Leaf, Mace. French Lavender, Long Pepper, Seeds of Harwort, Juice of the Rape of Cistus, Strained Storax, Opponax, Strained Galbanum, Balsam of Gilead, Oil of Nutmeg, Russian Castor, each .......... *eight* "
Water Germunder, Balsam-Tree Fruit, Cubeb, White Pepper, Seeds of Carrot of Crete, Poley Mont. Strained Bdellium, each .......... *seven* "
Gentian Root, Celtic Hard. Leaves of Dittany of Crete, Red Rose, Seeds of Macedonium Parsley, Sweet Fennel Seed, Seeds of Lesser Cardamom Gum Arabic, Odium, of each .......... *five* "
Sweet Flag, Wild Valerian, Anise Seed, Sagapernum, each .......... *three* "
Spigrul, St. John's Wort, Juice of Acacia, Catechu, Dried Bellies of Skunks. each .......... *two and one-half* "
Clarified Honey .......... 915 "

The roots, etc., to be finely powdered, and the whole mixed thoroughly.

"It is of importance to remember that this class of patients do not bear well the prolonged application of cold to the surface, either by means of the cold bath or the cold pack, and that, unless the anti-pyretic power of quinine is added to the application of cold, very little benefit will be obtained from the use of the latter. The danger resulting from the injudicious use of cold baths is greater in this than in any other infectious disease.

"The rules for the administration of stimulants are the same as those given for their administration in typhoid fever. The effects of the first few doses should be carefully watched. They should never be given indiscriminately, for there is greater danger in over-stimulating in this than in any other fever. Their use is indicated whenever signs of heart-failure are present, such as feeble pulse and an indistinct first sound of the heart. No fixed rule can be laid down as regards the quantity to be administered in any given case; it will vary with the type of the fever and the previous habits of the patient; it should always be administered at stated intervals. The period of the fever at which stimulants should be commenced will also vary. In some cases stimulants are never required, while in other cases, from the very outset of the fever, they are demanded. In the majority of cases their use is not indicated before the end of the second week. It must be borne in mind that alcohol is not a specific, curative agent in this fever, but that the object of its administration is to sustain the heart and prevent the vital powers from falling below the point at which reparative processes are possible. The use of stimulants is not necessarily contraindicated when delirium is present. Frequently after their administration the delirium will pass away, and only when it is decidedly increased by their use should they be abandoned.

"The state of the bowels, skin and kidneys demands the closest attention. If, early in the disease, the bowels are constipated, a calomel purge combined with ten or fifteen grains of quinine will often be followed by marked benefit. In any stage of the disease brisk purgation should be avoided. If diarrhœa is present, it should not be interfered with unless it becomes exhausting; then it should be checked by small doses of opium combined with astringents. Symptoms referable to disturbances of the nervous system sometimes require special treatment. If there is extreme restlessness, muscular twitchings, or active delirium, opium may be administered in full doses. The effect of the first dose must be carefully watched. If sleep soon follows its administration, and the delirium gradually subsides without any aggravation of the other symptoms, its use may be continued; if, instead of producing sleep, the patient becomes more wakeful, and the delirium is increased and more active, and the other symptoms are greatly aggravated, its use must be immediately abandoned. Under these circumstances chloral may be tried with great care.* Quain advises gr. xv.-xx. of bromide of potassium under similar conditions.

"Some claim that spirits of turpentine in the treatment of this form of fever has almost a specific power, while others regard it useful only as a stimulant. My own experience leads me to employ it only as a stimulant during the second and third week of the disease, when there is great prostration and marked typhoid symptoms. It may be given as an emulsion in doses of twenty drops every two hours. The *diet* best suited to patients with this fever is milk administered in the same way as was proposed in the case of typhoid fever patients. Special complications occurring during non-specific variety must be met with such remedies as the condition of the patient and the peculiar complications may require."

---

TRANSACTIONS OF THE MEDICAL ASSOCIATION OF GEORGIA —Thirty-fifth Annual Session, 1884. James P. Harrison & Co., Atlanta, Ga.: Publishers.

I regard this as perhaps, the most valuable issue of transactions in the history of the Association. Dr. James A. Gray, the Secretary, and the publisher, both deserve the thanks of the profession for the parts they have each so well maintained in the work; the

*Wood recommends Hoffman's anodyne and spts. æth. nitrosi for restlessness; and musk, asafœtida, camphor and similar drugs for the hiccough."

